# The Admission of Women to the London Colleges

**Published:** 1908-02-15

**Authors:** 


### The Hospital

A Journal of

The Medical Sciences and Hospital Administration.

New Series. No. 48, Vol. II. [No. 1120, Vol. XLIII.] SATURDAY, FEBRUARY 15, 1908.

### The Admission of Women to the London Colleges.

As already indicated in our columns, the long controversy in regard to the admission of women to the examinations of the London Colleges is reaching its final stage. The Committee of the Royal College of Physicians has decided by the overwhelming majority of 74 to 33 to admit women to all the examinations of the College, whilst the Council of the Royal College of Surgeons has passed a resolution "That it is desirable that women be admitted to examination for the diploma of Member," but has decided to take a poll of the Fellows and Members upon the question, and has appointed a committee to prepare a circular upon the matter. Presumably, therefore, the poll will shortly be taken; and if as a result the Council should decide still to exclude women, the College of Physicians, under the agreement between the two Colleges, will be unable to give effect to its resolution so far as the examination for its license is concerned.

It is therefore of considerable interest to consider what are the arguments for and against the admission of women to these examinations. It must be remembered that it is no longer a question of admitting them to the medical profession; that has been settled long ago, and it may surprise some to learn that nearly 800 women are now on the Medical Register and over 200 more studying medicine. They have proved their fitness for professional responsibility by the good work they have done not only in the East, work from which men are often debarred, but also in this country in hospitals for women and children, lunatic asylums, and other public institutions, and especially in connection with the rapidly increasing and most important work of medical inspection of school children. The public, as represented by many popularly elected bodies and by Government Departments, have not been slow to recognise the peculiar fitness of women for work of this kind; for instance, in the memorandum issued last November by the Board of Education in regard to the medical inspection of children, we find these significant words: "It should be noted that there are many cases in which women are likely to be specially suitable" (for appointment as medical inspectors). In view of the fact that women have thus gained a definite and permanent place in the professional life of the country, it surely behoves the English Colleges, as the most important licensing bodies in the kingdom, to take a proper share in examining them and so in directing their education, thus discharging a duty to the community from which is derived the responsibility of granting diplomas.

Once the position of women in the profession is recognised, it is difficult to see what valid arguments can be raised against their admission to the Colleges. It is sometimes asked why they are not satisfied with the many portals now open to them? The answer is simply that they wish to obtain the diplomas of the Colleges for precisely the same reasons that men do, and that it is a very real grievance, especially for those who desire to get their education in London, that they cannot qualify in the way that the great majority of male students do. For women, if unable to pass the examinations for the degrees of the University of London, have to be content with the License of the Society of Apothecaries. But perhaps the real feeling, unexpressed rather than avowed, which induces many practitioners to oppose the claims of women, is the fear that their competition will become increasingly serious. Fortunately, apprehensions on this score may confidently be set at rest, for no appreciable increase in the number of medical women is likely to result from this concession, so many qualifications being now available that no woman who wishes to study medicine is likely to be deterred by her inability to obtain the diplomas of the English Colleges. If these examinations are thrown open, what will probably happen will be that a certain number of women who would otherwise go to the Society of Apothecaries will take the M.R.C.S., L.R.C.P., that some will be content with the latter qualifications who would otherwise strive for the degrees of the University of London, and lastly, that a few students who, as things are at present, would receive their education in the provinces, in Scotland, or in Ireland, will be induced to come to London instead. Few, indeed, will be those who will enter the medical profession merely because the London Colleges are open to them.

It is to be hoped, therefore, that even those medical men who still entertain a certain sentiment or prejudice against medical women will recognise the position they have already attained in the face of great obstacles and realise that large numbers of them are working harmoniously with men in public institutions of all kinds, that many are in practice in one or other of nearly all the larger towns of the United Kingdom, and that others are doing most valuable and self-sacrificing work, often at great risk to life and health, in Eastern countries. Moreover, those practitioners who are brought most intimately into contact with medical women and see most of their work are almost invariably their enthusiastic supporters. If these facts are once thoroughly understood, we believe that the great majority of the Fellows and Members of the College of Surgeons will by their votes at the approaching poll help to remove what is almost the last disability under which medical women still labour. It would be little less than a scandal were the general practitioners of the country to fail to endorse the deliberately expressed opinion of the governing bodies of the Colleges on this question.

